# Adjustable Parameters and the Effectiveness of Adjunct Robot-Assisted Gait Training in Individuals with Chronic Stroke

**DOI:** 10.3390/ijerph19138186

**Published:** 2022-07-04

**Authors:** Shih-Ching Chen, Jiunn-Horng Kang, Chih-Wei Peng, Chih-Chao Hsu, Yen-Nung Lin, Chien-Hung Lai

**Affiliations:** 1 Department of Physical Medicine and Rehabilitation, School of Medicine, College of Medicine, Taipei Medical University, Taipei 110, Taiwan; csc@tmu.edu.tw (S.-C.C.); jhk@tmu.edu.tw (J.-H.K.); 2 Department of Physical Medicine and Rehabilitation, Taipei Medical University Hospital, Taipei 110, Taiwan; joehsu1986@gmail.com; 3 School of Biomedical Engineering, College of Biomedical Engineering, Taipei Medical University, Taipei 11031, Taiwan; cwpeng@tmu.edu.tw; 4 School of Gerontology Health Management, College of Nursing, Taipei Medical University, Taipei 110, Taiwan; 5 Institute of Biomedical Engineering, National Taiwan University, Taipei 10617, Taiwan; 6 Graduate Institute of Injury Prevention and Control, Taipei Medical University, Taipei 110, Taiwan; semitune@gmail.com; 7 Department of Physical Medicine and Rehabilitation, Wan Fang Hospital, Taipei Medical University, Taipei 11696, Taiwan; 8 Taipei Neuroscience Institute, Taipei Medical University, Taipei 110, Taiwan

**Keywords:** stroke, robot-assisted gait orthosis, motricity index, Barthel Index, guidance force, body-weight support

## Abstract

The aims of this study were (1) to compare the effect of robot-assisted gait orthosis (RAGO) plus conventional physiotherapy with the effect of conventional therapy alone on functional outcomes, including balance, walking ability, muscle strength, daily activity, and cognition, in chronic stroke patients, and (2) to determine the association of adjustable parameters of RAGO on functional outcomes. Adjustable parameters of RAGO included guidance force, treadmill speed, and body-weight support. This retrospective cohort study enrolled 32 patients with chronic stroke. Of these, 16 patients received RAGO plus conventional physiotherapy (RAGO group), and 16 patients received conventional physiotherapy alone (control group). Balance was assessed using the Berg Balance Scale, walking ability using the Functional Ambulation Category, muscle strength using the Motricity Index, daily activity using the Barthel Index, and cognition using the Mini-Mental State Examination. The scores were assessed before and after training. The Mini–Mental State Examination and the Berg Balance Scale increased significantly in both groups, whereas improvements in the Motricity Index and the Barthel Index were only observed in the RAGO group after intervention. During RAGO training, reducing guidance force and body-weight support assistance was associated with improvements in the Barthel Index, whereas higher treadmill walking speed was associated with improvements in the Berg Balance Scale. Our study found that RAGO combination therapy resulted in improvements in more functional outcomes than did conventional training alone. The adjustable parameters of the RAGO training were partly associated with training outcomes.

## 1. Introduction

Stroke is a leading cause of serious, long-term disability in adults [1,2]. Walking dysfunction and residual gait impairment are common after stroke, which consequently affect mobility and daily living activities [3,4]. Hence, for people with stroke, therapies to restore walking ability, gait function, and independence are essential.

A wide range of post-stroke hemiplegic gait impairments is clinically observed, which are consequences of impaired swing initiation and single limb support in the paretic limb and related compensatory strategies [5]. More recently, Li et al., 2018, proposed that post-stroke hemiplegic gait impairments could be mechanical consequences of altered neural control mechanisms of human gait and that muscle weakness, spasticity, and spastic activation on the paretic trunk and leg need to be taken into consideration during clinical evaluation and designing of rehabilitation programs [4].

Conventional gait therapy and body-weight–support treadmill training remain the most commonly used therapeutic interventions. These methods apply one of the following four approaches: (1) Aggressive mobilization using a brace, walking assist device, and physical assistance by the therapist; (2) The Brunnstrom technique, encouraging the use of synergistic movements; (3) Proprioceptive neuromuscular facilitation, encouraging the use of spiral and diagonal movements; or (4) Neuro-developmental therapy using reflex inhibitory movements [6]. Literature has demonstrated that four weeks of conventional rehabilitation had a positive impact on maximal gait speed and the Motricity Index in individuals with stroke [7]. Body-weight–support treadmill training demonstrated improved walking speed and endurance but there was no obvious advantage over conventional gait training for persons with chronic stroke [8]. A study by Druhbicki et al., 2018 also demonstrated that gait training using body-weight-support treadmill, with or without visual biofeedback, improved spatiotemporal gait parameters, walking speed, endurance, and mobility in individuals with subacute stroke [9]. While these interventions have proven effective for improving gait and functional recovery after stroke [10], their use may be limited by practical considerations, as they often require two or more therapists to control the paretic limbs and trunk during training [6,10]. 

Robot-assisted gait orthosis (RAGO), which provides mechanical assistance to limb movement during body-weight–support treadmill training [11], allows for safe and vigorous repetitive training without the labor-intensive assistance of a physical therapist required in conventional physiotherapy [12], as well as precise movement control and instant modification of movements using biofeedback [13,14]. 

While previous studies on the efficacy of RAGO show that patients benefit from this method, whether these benefits exceed those achieved by conventional physiotherapy remains controversial. One study reported that RAGO facilitates plasticity in the intact supplementary motor area of the affected hemisphere, demonstrating that this method has added value to motor recovery over that of conventional physiotherapy [15]. A meta-analysis showed that RAGO improves balance in individuals with stroke, but data comparing these improvements with those of other gait rehabilitation methods are inconsistent [16]. In patients at the subacute stage of stroke, some studies have reported similar effectiveness between RAGO training and conventional physiotherapy for walking speed and performance [17,18], whereas others have reported the superiority of RAGO training in increasing functional ambulation [19], improving gait symmetry [19] and the advantages of conventional gait training on walking speed and distance [20]. Some studies suggest that RAGO training in combination with conventional physiotherapy may yield superior effects on walking ability to those of conventional physiotherapy alone [21,22]. In contrast, for patients at the chronic stage of stroke, evidence suggests that therapist-assisted intervention provides greater improvements than RAGO training with regard to walking ability [23,24]; however, this difference has not been observed consistently between studies [25,26]. In fact, another study reports RAGO training to be more effective than treadmill gait training in improving walking ability and balance in patients with chronic stroke [27]. 

Given these widely varying results, whether the positive effects of RAGO training are greater than those of conventional physiotherapy in patients with chronic stroke remains unclear. The finding in some studies that RAGO yields worse outcomes than conventional physiotherapy might be explained by the constraints of robotic-assisted training, which provides gait training in one fixed direction, contrasted to that of therapist-assisted physiotherapy. Another potential source of the differing outcomes of previous RAGO studies is the parameter settings chosen for the robotic device. These adjustable parameters, which include guidance force, treadmill speed, and body-weight support, affect the interaction between the robotic device and its wearer and, thus, may influence the outcomes of RAGO training. While few studies have examined the relationship between RAGO parameter settings and training outcomes, Knaepen et al., 2015 demonstrated that guidance force during gait training was associated with sensorimotor cortex activity, which is crucial for motor learning [28]. However, most of the previous RAGO studies have focused on its effects on gait performance. 

The objectives of this retrospective cohort study were (1) to compare the effect of RAGO plus conventional physiotherapy with the effects of conventional therapy alone on functional outcomes, including balance, walking ability, muscle strength, daily activity, and cognition; and (2) to determine the association of adjustable parameters of RAGO, including guidance force, treadmill walking speed, and body-weight support on studied functional outcomes (balance, walking ability, muscle strength, daily activity, and cognition). Balance was evaluated using the Berg Balance Scale, walking ability using the Functional Ambulation Category, muscle strength using the Motricity Index, daily activity using the Barthel Index, and cognition using the Mini-Mental State Examination. The differences in these outcomes before and after training were compared. We hypothesize that patients receiving RAGO plus conventional therapy would benefit from conventional physiotherapy alone. In addition, we propose that the adjustable parameters of RAGO might be associated with the functional outcomes. 

## 2. Methods

### 2.1. Patients

The medical records of consecutive patients with chronic stroke (≥6 months after onset) who received rehabilitation in the Department of Physical Medicine and Rehabilitation, Taipei Medical University Hospital, between July 2010 and June 2016, were reviewed, retrospectively. The inclusion criteria for RAGO and conventional training groups were (1) first-time stroke, according to the World Health Organization criteria used at the hospital; (2) a single lesion confirmed using Computed Tomography or Magnetic Resonance Imaging; (3) limbs with hemiplegia or hemiparesis; (4) Brunnstrom stage between II to V; and (5) completion of 12 sessions of conventional physiotherapy, either alone or in combination with RAGO training at Taipei Medical University Hospital. The exclusion criteria were (1) failure to complete all 12 sessions of RAGO training combined with physiotherapy or conventional physiotherapy alone, (2) incomplete outcome assessment data, and (3) failure to complete 3 60 min physiotherapy sessions per week, in the conventional physiotherapy group. Additionally, at our institute, individuals are not offered RAGO intervention if they have (1) a body weight > 130 kg, (2) weight-bearing difficulty due to unstable fractures or severe osteoporosis, (3) skin problems severe enough to preclude the use of the Lokomat harness, (4) orthostatic hypotension, (5) an unstable medical condition, (6) severe joint contracture, (7) severe vascular disorders of the lower limbs, or (8) a psychological disorder. 

This study was conducted at Taipei Medical University Hospital, and the study protocol was reviewed and approved by the Joint Institutional Review Board of Taipei Medical University (TMU-JIRB-N201510055).

### 2.2. Study Groups

**Figure 1:**
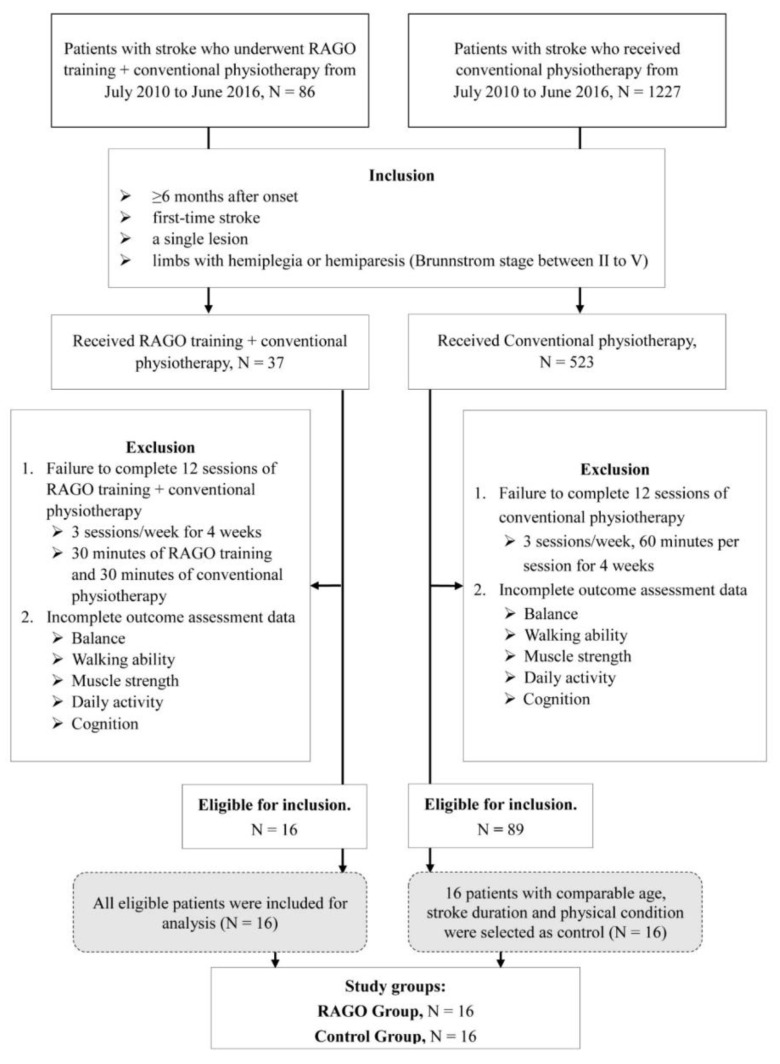
Study flow chart. Abbreviation: RAGO, robot-assisted gait orthosis.

This is a retrospective cohort study. The flow chart of patients included in the study is shown in Figure 1. Patients who received RAGO plus conventional physiotherapy were defined as the RAGO group, and patients who received conventional physiotherapy alone were defined as the Control group.

The RAGO group included 16 patients with chronic stroke (mean time from onset, 20.64 ± 4.83 months). The control group included 16 chronic stroke patients who received conventional poststroke physiotherapy alone (mean time from onset, 19.31 ± 6.57 months). The control group had comparable age, stroke duration, and physical condition (Brunnstrom stage, mobility on level surface) to the RAGO group. Patients with incomplete training sessions, or incomplete outcome assessment data, were excluded.

### 2.3. Treatment

The control group received 60 min of conventional poststroke physiotherapy. Conventional physiotherapy involved tasks such as rolling, sitting, balance exercises, standing, overground walking, and paretic limb facilitation. Therapists adjusted the therapy according to the functional status of the patient.

RAGO training was performed on a Lokomat system (Hocoma AG, Volketswil, Switzerland). RAGO training sessions were incorporated into conventional poststroke physiotherapy. The patients received 12 training sessions (3 sessions/week for 4 weeks) that included 30 min of RAGO training and 30 min of conventional physiotherapy (in no particular order), with no break in between except for the time required for the system setup. RAGO requires a 10-min period for setup and calibration of the Lokomat training system (excluded from the 30-min training time). Therefore, the total training time was 60 min.

The RAGO training used a body-weight support system to support body weight, electromechanical gait orthosis to drive legs on the sagittal plane of the treadmill, a mirror, and a computer to display the speed, force, and bilateral hip and knee motion to provide visual feedback regarding the gait pattern (Supplement Figure S1). Physiotherapists provided verbal interaction and visual feedback using the parameters on the screen to remind the patient to move both the paretic and healthy leg to achieve as symmetrical a reciprocal gait as possible. The physiotherapists adjusted the parameters manually and could adjust them in real-time during training. They sometimes provided additional manual assistance to the paretic leg during the stance and swing phases if the patient was losing concentration or not expending enough effort. 

### 2.4. Outcome Measures

Primary outcome was muscle strength (lower extremity) assessed using the Motricity Index. Secondary outcomes were: balance (assessed using the Berg Balance Scale), walking ability (using the Functional Ambulation Category), independence in daily activity (using the Barthel Index), and cognition (using the Mini–Mental State Examination). Measurements were collected from the medical records. Before training measurement (t = 0) was obtained one day before session 1. After training measurement (t = 1 to t = 12) was measured 1 day after the completion of each session. A total of 12 training sessions affected the outcome measures. 

#### 2.4.1. Mini–Mental State Examination 

The Mini–Mental State Examination is a frequently used, and easily applied, instrument in evaluating cognitive status [29,30]. It evaluates cognitive functions, i.e., immediate memory, orientation, registration/delayed recall, attention/concentration, and language. It has a maximum score of 30, with higher scores representing better cognitive function.

#### 2.4.2. Berg Balance Scale

The Berg Balance Scale assesses balance and risk of falls. It is a series of 14 functional balance tasks. Each task is scored on a 5-point ordinal scale, with a score of 0 denoting inability to perform the task and that of 4 denoting the ability to fully complete the task using preset criteria. The highest possible score is 56 [31,32,33]. In older adults and patients with stroke, Berg Balance Scale has high inter-rater and intra-rater reliability [34].

#### 2.4.3. Motricity Index

The Motricity Index is a reliable index for evaluating paralysis in patients with stroke. It is used to measure motor function for the entire motor function system of the affected upper and lower extremities and has a maximum index value of 100. In this study, we used the Motricity Index to assess lower extremity strength [35]. The scoring of lower extremity muscle action strength using the Motricity Index were 0 (No movement), 9 (Palpable contraction, but no movement), 14 (Movement, but not full range or against gravity), 19 (Movement, full range against gravity, not against resistance), 25 (Movement against resistance, weaker than the contralateral side) and 33 (Normal strength).

#### 2.4.4. Functional Ambulation Category

The Functional Ambulation Category is used to classify the degree of walking ability after stroke. The 6 categories in the Functional Ambulation Category were: 0 (non-ambulatory), 1 (needs continuous support from 1 person), 2 (needs intermittent support from 1 person), 3 (needs only verbal supervision), 4 (help is required on stairs and uneven surfaces), and 5 (can walk independently anywhere) [36].

#### 2.4.5. Barthel Index

The Barthel Index is a 10-item measurement of independence in activities of daily living. It was used to assess functional changes after stroke [37]. The scale ranged from 0 to 100, and was assessed according to the amount of assistance required by the patient. Lower scores represent greater nursing dependency. A score of less than 40 tends to predict a lack of independence in motor skills and difficulty with other basic skills, whereas scores over 60 tend to demonstrate a transition from dependence to assisted independence.

#### 2.4.6. Brunnstrom Stage

The Brunnstrom stage is the sequence of motor development and reorganization of the brain after stroke. The range of the Brunnstrom stage includes flaccidity (stage 1), spasticity appearance (stage 2), increased spasticity (stage 3), decreased spasticity (stage 4), minimal spasticity (stage 5), and normal movement with normal speed (stage 6) [38]. Brunnstrom stage data before and after training were compared to assess stroke recovery.

### 2.5. Adjustable RAGO Parameters

Adjustable RAGO parameters, including guidance force, treadmill speed, and body-weight support, were used as measurable outcomes [39]. A guidance force of 100% provides 100% of the movement force, such that the limb is moved only by the machine. This guidance force was gradually decreased at intervals of 5% to encourage active movement by the patient. Walking speed started at an initial speed of 0.42 m/s in the initial session and increased gradually as tolerated to a maximum speed of 0.89 m/s [39]. The body-weight support initially provided approximately 40% support to each participant and was decreased such that the active force exerted by the patient required effort but was not impossible. These three parameters were adjusted according to the real-time performance of the patients during RAGO training, depending on whether the defined physiological or task-oriented gait pattern was achieved.

### 2.6. Sample Size

The sample size was determined based on the effect size of two prior studies. A cross-over study by Mayr et al., 2007 (n = 16 stroke subjects) demonstrated significantly improved walking ability and walking speed during Lokomat intervention periods [17]. Another study by Chisari et al., 2015 (n = 15 stroke patients), showed that the Berg Balance Scale, time up and go test, and 6-min walking test were significantly improved after Lokomat training [40]. A priori power analysis was performed using an algorithm for estimation of power for mean difference with two independent means in G*Power software v 3.1. The minimum required sample size necessary to detect a significant difference with statistical power of 80% was n = 15 per group. Assuming an expected effect size d was 1.0667, a total of 30 samples was required to reach power of 0.8. Therefore, a total of 32 patients were included in our study.

### 2.7. Statistical Analysis

Variables were measured before the first, and after the last, session (1 day before training of session 1 and 1 day after the end of session 12), and outcomes were based on the difference between before session 1 and after session 12. For this study, before training measurements (t = 0) were measured 1 day before training of session 1; After training measurements (t = 1) were measured 1 day after the completion of session 12.

Continuous variables are presented as the mean ± SD, whereas nominal variables are presented as frequencies and percentages. Due to nonnormal data distribution, the between-group differences in baseline characteristics and study outcomes were compared using the Mann-Whitney U test for continuous variables; the within-group comparison of study outcomes pre- and post-training was determined using the Wilcoxon signed-rank test. Repeat-measure ANOVA was performed to evaluate the changes in adjustable parameters of the RAGO (guidance force, treadmill walking speed, and body-weigh support) between each RAGO training session. The effect of time on the variables of adjustable RAGO parameters in the RAGO group as well as the effect of time and group on the functional outcomes of both groups were also analyzed by repeated measurement ANOVA. Spearman’s correlation coefficients were calculated to assess the relationship between study outcomes and adjustable parameters of the RAGO (guidance force, walking speed, and body-weight support). The magnitudes used to interpret the correlation coefficients were as follows: (1) |r| < 0.1, trivial; (2) 0.1 ≤ |r| ≤ 0.29, weak; (3) 0.3 ≤ |r| ≤ 0.49, moderate; and (4) |r| ≥ 0.5, strong [41]. All statistical analyses were performed using SAS (version 9.4; SAS Institute, Cary, NC, USA). Statistical significance was considered as a 2-tailed *p* < 0.05.

## 3. Results

### 3.1. Baseline Characteristics of Patients

Patient baseline characteristics, including sex, age, side of stroke, stroke type, stroke duration, and physical condition, are shown in Table 1. The results showed that the distribution of baseline characteristics was similar between groups (all *p* > 0.05). The physical condition of stroke (mobility on level surface, and Brunnstrom stage) before training were comparable between the two groups (all *p* > 0.05) suggesting the two groups had comparable functional status at baseline.

### 3.2. Outcome Variables before and after Training

The main outcome measurements before and after training are shown in Table 2. The effect of time and group on the functional outcomes in Table 2 are exhibited in Table 3. After training, both groups demonstrated a significant increase in the Berg Balance Scale, and Mini–Mental State Examination scores. These results implied that both RAGO and control groups improved balance and cognition after intervention. The RAGO group demonstrated additional significant increases in Motricity Index of lower extremity, Barthel Index, and mobility on level surfaces. These findings suggested that a combination of RAGO and conventional physiotherapy additionally improved lower extremity muscle strength, independence in daily activities, and mobility on level surfaces after training. In summary, the above findings suggested that a combination of RAGO and conventional physiotherapy had intra-group benefits compared to conventional physiotherapy alone.

The Brunnstrom stages of patients before and after training are shown in Table 4. Before training, the upper extremity and lower extremity Brunnstrom stages were similar between the control and RAGO groups (*p* > 0.05). After training, both groups showed a trend of improvements in the lower extremity-Brunnstrom stage (*p* = 0.06 and 0.08, respectively). This suggested improvement in terms of stroke recovery stage in patients receiving either the control or RAGO therapy.

### 3.3. Robot-Assisted Gait Orthosis (RAGO) Training Adjustable Parameters

**Figure 2:**
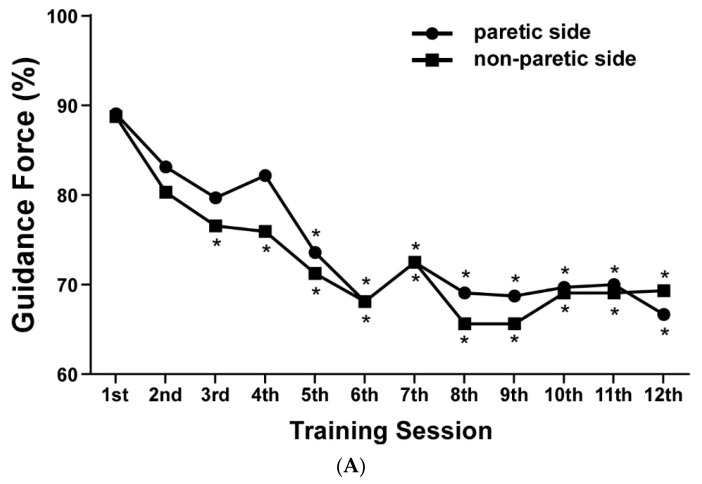

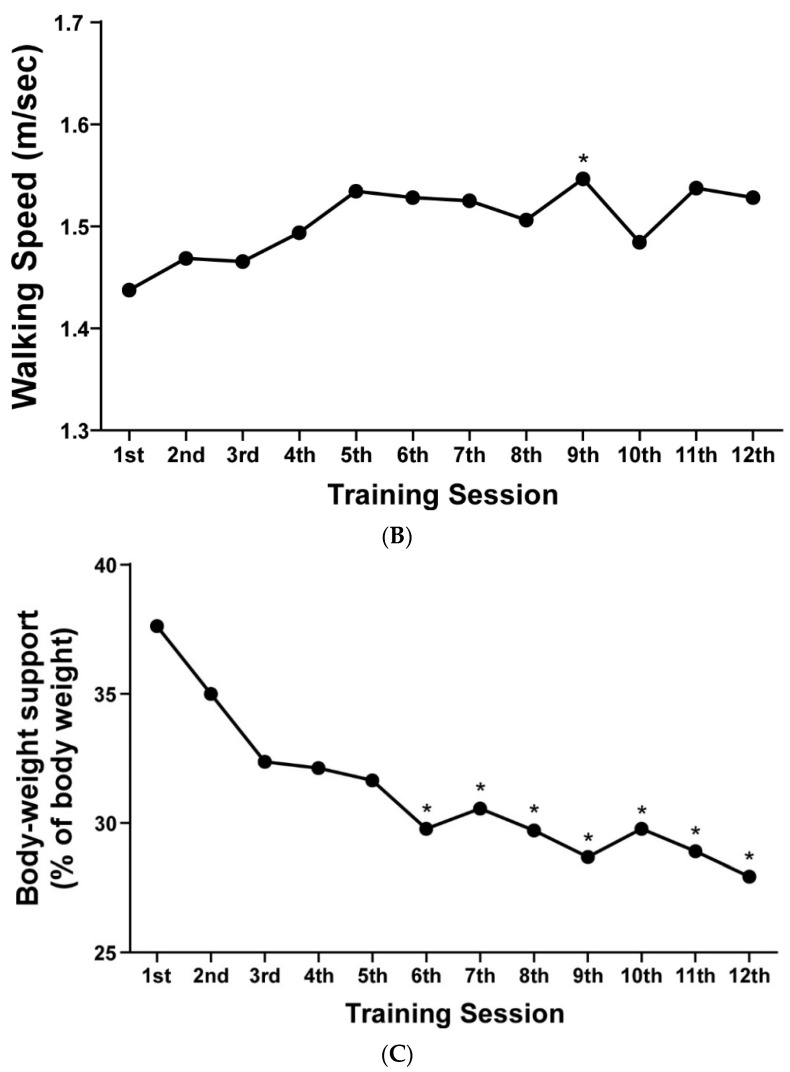
Robot-assisted gait orthosis (RAGO) training parameter trends: (**A**) guidance force; (**B**) walking speed; and (**C**) body-weight support for patients in the RAGO group. * p < 0.05 according to repeated-measures analysis of variance.

The effect of time on the variables of adjustable RAGO parameters are shown in Table 5. From sessions 5 to 12, the guidance force required on the paretic side was significantly lower than that in the first session on the paretic side (Figure 2A). The decrease in guidance force in sessions 5 to 12 implied that paretic limbs required less assistance of external force and increased muscle contraction during sessions 5 to 12 of RAGO training. An increasing trend in improved treadmill walking speed was observed. A significantly fast speed was achieved in session 9 compared to that in session 1 (Figure 2B). The body-weight support required was significantly lowered in sessions 6 to 12 than that required in session 1 (Figure 2C). These findings suggested that lower extremities increased muscle activity and the ability of body-weight support during sessions 6 to 12 of RAGO training.

### 3.4. Association between RAGO Parameters and Functional Outcome Variables

The association between functional outcome measurements and adjustable RAGO parameters is shown in Table 6. The Barthel Index strongly correlated with changes in guidance force and body-weight support in both legs, and the Berg Balance Scale was strongly correlated with changes in treadmill speed. These results implied that lower guidance force and less body-weight support assistance were associated with large increases in daily activity independence, whereas high treadmill walking speed was associated with large increases in balance status. This suggested the change of adjustable RAGO parameter conditions during training was partly associated with improvement of functional outcomes.

## 4. Discussion

Our results indicated a significant improvement in balance, and cognition in both groups after training. However, significant improvements in lower extremity muscle strength, daily activity independence, and mobility on level surfaces were only observed for the RAGO group. Thus, this study suggests that RAGO training may potentially play- a complementary role in the training of chronic stroke.

RAGO training combined with conventional physiotherapy may be more effective for several reasons. Patients receiving RAGO training perform many highly repetitive movements and undergo precise walking pattern training. Such patterning might be more predictable and consistent than that provided by therapists [42]. RAGO provides sagittal plane assistance to hip and knee joint movements that approximate a symmetrical reciprocal gait. The physiotherapist sometimes provides additional manual assistance to the affected limb during the stance and swing phases. As the walking speed gradually increases, as tolerated by the patient, the guidance force or the amount of assistance provided by RAGO training to move the legs through the sagittal plane decreases. Initially, 100% guidance force is supplied for both legs and is then gradually reduced as the patient’s ability to perform active movements improves. This prevented the patients from over-reliance on robotic assistance. The decrease in guidance force encouraged the patients to exert active effort, and cued more active muscle contractions during RAGO training. Although a study found that muscles remain active in controlling leg movement even if full guidance is provided [43], Krishnan et al., 2013 reported that the decreasing need for guidance over the course of robotic training indicates that patient-cooperative robotic training facilitates gait recovery after stroke [44]. We observed that body-weight support gradually decreased below that of the first session and was significantly lower than the first session by the sixth session. Van Kammen et al., 2016 reported that leg muscle activity increased with decreasing guidance force and body-weight support [43]. Therefore, intensive and highly repetitive walking training involves active muscle contraction and may increase lower leg muscle strength and motor learning [45], as reflected by the improvement in the Motricity Index and the subsequent improvement in the Barthel Index. Tyson et al., 2018 also reported that traditional post-stroke therapy for balance and mobility problems typically uses low-dose, low intensity, and therapist-led practice of functional tasks and suggested that therapists had to maximize the intensity of functional task practice [46]. However, RAGO training alone does not include several activities important for the generalization of motor learning i.e., postural control tasks and stepping over objects [42]. Therefore, combining RAGO with conventional physiotherapy, that encompasses activities such as rolling, sitting, balance exercises, standing, postural tasks, and overground walking, is crucial for individuals with chronic stroke.

Few studies have investigated the association between the adjustable parameters of the RAGO training environment and outcome measures after RAGO training. The results of the present study suggest that the lower the guidance force or the less the body-weight support assistance, the greater the Barthel Index changes. In one case report, lower guidance force was observed to facilitate gait function in a stroke survivor [44]. In a study of 10 patients with subacute stroke, Lee et al., 2017 found that guidance force affected oxygen consumption at certain gait speeds [47]. Knaepen et al., 2015 demonstrated that guidance force during gait training was associated with sensorimotor cortex activity, which was demonstrated as being crucial for motor learning [28]. The same study suggested that higher motor learning and gait function resulted in greater changes in the Barthel Index. Similarly, our findings indicated that the higher the treadmill walking speed, the greater the changes in the Berg Balance Scale. In a recent study of 10 patients with stroke, van Kammen et al., 2019, reported that the only parameter to significantly affect muscle activity was speed [48]. Another study by McGrath et al., 2019 also showed that gait speed had a significant effect on the profiles in all joints of the lower extremities in healthy adults [49]. Together with our findings, these results suggest that gait speed increases muscle activity and joint moment, resulting in improvements in the Berg Balance Scale.

### Strengths and Limitations

This study has several limitations. Due to the small sample size, the results should be regarded as preliminary findings, which may not be representative of all patients undergoing RAGO combination training. Further prospective study is required to validate the present finding. Secondly, this study was retrospective in nature, and some bias in forming the two groups may not have been accounted for. For example, RAGO training required payment by the patient while conventional physiotherapy alone did not. In addition to stratifying patients, based on ability to pay, this factor may have had a psychological effect on the response to training. However, we have shown in tables that the two groups had comparable baseline characteristics (no statistically significant differences between the RAGO combination training group and conventional control group) in age, sex, stroke type, stroke duration, mobility on level surface, Brunnstrom stage score, and baseline outcome measures before intervention. Thirdly, this study only examined the effectiveness of intervention immediately post-training. Long-term effects of rehabilitation may be different in stroke survivors and warrant further investigation.

Despite these limitations, this study is one of the few that investigated the association between RAGO parameter settings and a wide variety of functional outcomes. Another strength of this study was the use of a wide variety of outcome measurements, including cognition, balance, muscle strength, walking ability, and daily activity independence to compare the efficacy of combination RAGO training and conventional physiotherapy with conventional physiotherapy alone.

## 5. Conclusions

Patients with chronic stroke can attain improvements in cognition, balance status, and walking ability after conventional physiotherapy alone or RAGO training in combination with conventional physiotherapy. The significant improvements in clinical outcome measures of the Barthel Index and the Motricity Index in patients undergoing RAGO combined with conventional physiotherapy suggest that the combination approach may have additional benefits for improvements in lower limb strength and daily activity independence in patients with chronic stroke. In addition, our study suggests adjustments of RAGO parameter settings during training were partly associated with training outcomes. Reducing guidance force and body-weight support assistance during RAGO training was related to greater improvements in Barthel Index scores, and increased treadmill walking speed during RAGO intervention was related to greater improvements in Berg Balance Scale. The mechanisms underlying these observations and the long-term effects of RAGO training warrant further investigation. Moreover, these results should be regarded as preliminary findings. It is necessary to further validate them by longer interventions and larger samples.

## Figures and Tables

**Table 1:** Patient Baseline Characteristics.

	Control	RAGO	*p*-Value
	N = 16	N = 16	
**Sex**			
** Male**	9 (56.25%)	8 (50.0%)	0.72 ^a^
** Female**	7 (43.75%)	8 (50.0%)	
**Age, years**	58.0 (16.1)	59.8 (14.1)	0.59 ^†^
** ≥65**	5 (31.25%)	6 (37.5%)	0.71 ^a^
**Side of stroke**			
** Left**	6 (37.5%)	10 (62.5%)	0.15 ^a^
** Right**	10 (62.5%)	6 (37.5%)	
**Stroke type**			
** hemorrhagic**	6 (37.5%)	10 (62.5%)	0.15 ^a^
** ischemic**	10 (62.5%)	6 (37.5%)	
**Stroke duration, months**	18.9 (26.1)	19.3 (13.3)	0.26 ^†^
**Brunnstrom stage**			
** Upper extremity**	3.4 (1.21)	3.7 (1.34)	0.53 ^†^
** Lower extremity**	3.5 (0.89)	3.8 (0.84)	0.37 ^†^
**Mobility on level surface**			
** 0**	5 (31.25%)	3 (18.75%)	0.58 ^†^
** 5**	3 (18.75%)	3 (18.75%)	
** 10**	3 (18.75%)	5 (31.25%)	
** 15**	5 (31.25%)	5 (31.25%)	

RAGO, robot-assisted gait orthosis. Level of impairment was assessed using the Barthel Index for Activities of Daily Living subscale: Mobility on level surfaces. Data representing number (%), or mean (SD); ^a^ Chi-square test or Fisher’s exact test. ^†^ Mann-Whitney U test.

**Table 2:** Functional Outcomes Before and After Training.

	Control	RAGO	Mean Difference (95% CI) ^¶^	*p*-Value ^†^
	N = 16	N = 16		
**Muscle Strength (Motricity Index of lower extremity)**				
Before training	17.2 (9.50)	19.1 (5.42)	−1.87 (−7.52, 3.77)	0.62
After training	18.2 (9.60)	21.8 (4.89)	−3.62 (−9.20, 1.95)	0.31
*p*-value ^‡^	0.36	0.04 *		
Difference	1.0 (4.15)	2.7 (4.42)	−1.75 (−4.84, 1.34)	0.21
**Balance (Berg Balance Scale)**				
Before training	25.8 (21.88)	22.3 (16.44)	3.50 (−10.47, 17.47)	0.74
After training	29.4 (22.84)	28.3 (16.89)	1.12 (−13.43, 15.68)	0.91
*p*-value ^‡^	0.02 *	<0.0001 *		
Difference	3.6 (5.82)	5.9 (4.43)	−2.37 (−6.11, 1.36)	0.03 *
**Daily Activity Independence (Barthel Index)**				
Before training	56.9 (38.38)	57.5 (28.58)	−0.62 (−25.05, 23.80)	0.95
After training	59.7 (36.76)	63.4 (25.61)	−3.75 (−26.74, 19.24)	0.88
*p*-value ^‡^	0.42	0.02 *		
Difference	2.8 (9.99)	5.9 (8.21)	−3.12 (−9.72, 3.47)	0.29
**Mobility on level surfaces**				
Before training	7.5 (6.33)	8.8 (5.63)	−1.25 (−5.57, 3.07)	0.58
After training	8.1 (6.55)	10.9 (5.23)	−2.81 (−7.09, 1.46)	0.19
*p*-value ^‡^	0.16	0.04 *		
Difference	0.6 (1.70)	2.2 (4.07)	−1.56 (−3.86, 0.73)	0.19
**Cognition Function (Mini–Mental State Examination)**				
Before training	21.3 (8.96)	21.6 (8.07)	−0.25 (−6.40, 5.90)	0.91
After training	22.7 (8.07)	23.6 (7.92)	−0.87 (−6.64, 4.89)	0.39
*p*-value ^‡^	0.03 *	0.02 *		
Difference	1.4 (2.28)	2.0 (3.31)	−0.62 (−2.67, 1.42)	0.21
**Walking Ability (Functional Ambulation Category)**				
Before training	2.7 (1.85)	2.2 (1.60)	0.50 (−0.75, 1.75)	0.44
After training	3.1 (2.00)	2.5 (1.71)	0.62 (−0.71, 1.96)	0.29
*p*-value ^‡^	0.06	0.06		
Difference	0.4 (0.89)	0.3 (0.48)	0.12 (−0.39, 0.64)	0.82

RAGO, robot-assisted gait orthosis; CI, confidence interval; Mobility on level surfaces: Barthel Index for Activities of Daily Living subscale. Time of measurements: Before training (t = 0), 1 day before starting session 1; After training (t = 1), 1 day after the completion of session 12. Difference = After-training score − Before-training score. ^†^ Mann-Whitney U test. ^‡^ Wilcoxon signed rank test. * Statistically significant (*p* < 0.05). ^¶^ Mean difference, control vs. RAGO groups.

**Table 3:** The effect of time and group on the functional outcomes in Table 2.

	F Statistics	*p*-Value
**Muscle strength (lower extremity)**		
Group effect	1.11	0.3
Time effect	6.13	0.02
Time x Group effect	1.33	0.26
**Balance**		
Group effect	0.11	0.74
Time effect	26.98	0.00001
Time x Group effect	1.69	0.2
**Daily activity independence**		
Group effect	0.04	0.85
Time effect	7.32	0.01
Time x Group effect	9.34	0.34
**Mobility on level surfaces**		
Group effect	4.83	0.05
Time effect	3.00	0.10
Time x Group effect	0.002	0.97
**Cognition**		
Group effect	0.04	0.84
Time effect	11.31	0.002
Time x Group effect	0.39	0.54
**Walking ability**		
Group effect	0.82	0.37
Time effect	8.78	0.01
Time x Group effect	0.24	0.63

Functional outcomes in Table 2, analyzed by Repeated measurement ANOVA. Time x Group effect is the interaction effect between two groups and over time.

**Table 4:** Brunnstrom Stage Before and After Training.

Brunnstrom Stage	Control N = 16	RAGO N = 16	Mean Difference (95% CI) ^¶^	*p*-Value ^†^
**Upper extremity**				
Before training	3.4 (1.21)	3.7 (1.34)	−0.31 (−1.23, 0.61)	0.53
After training	3.7 (1.24)	4.0 (1.28)	−0.31 (−1.22, 0.60)	0.49
*p*-value ^‡^	0.06	0.03 *		
Difference	0.3 (0.60)	0.3 (0.54)	0.05 (−0.39, 0.49)	0.79
**Lower extremity**				
Before training	3.5 (0.89)	3.8 (0.84)	−0.31 (−0.93, 0.31)	0.37
After training	3.9 (0.81)	4.0 (0.99)	−0.18 (−0.84, 0.46)	0.52
*p*-value ^‡^	0.08	0.06		
Difference	0.4 (0.80)	0.3 (0.51)	0.12 (−0.41, 0.66)	0.52

RAGO, robot-assisted gait orthosis; CI, confidence interval. Time of measurements: Before training (t = 0), 1 day before starting session 1; After training (t = 1), 1 day after the completion of session 12. Difference = After-training score − Before-training score. ^†^ Mann-Whitney U test. ^‡^ Wilcoxon signed rank test. * Statistically significant (*p* < 0.05). ^¶^ Mean difference, control vs. RAGO group.

**Table 5:** The effect of time on the variables of adjustable RAGO parameters.

	F Statistics	*p*-Value
Guidance force—Paretic side	10.36	<0.0001
Guidance force—Non-paretic side	10.36	<0.0001
Walking speed	2.95	0.02
Body-weight support	7.11	<0.0001

Adjustable parameters of RAGO were analyzed by Repeated measurement ANOVA.

**Table 6:** Correlation Between Outcome Measures and RAGO Parameters in Patients with Stroke.

	Guidance Force (Paretic Side)	Guidance Force (Non-Paretic Side)	Speed	Body-Weight Support
Muscle strength (Motricity Index of lower extremity)	0.199	0.192	0.092	−0.231
Walking ability (Functional Ambulation Category)	0.099	0.099	0.284	−0.168
Daily activity independence (Barthel Index)	−0.644 **	−0.613 *	−0.172	−0.634 **
Cognition (Mini–Mental State Examination)	0.247	0.175	0.181	0.054
Balance (Berg Balance Scale)	−0.008	−0.006	0.601 *	−0.023

Data are presented as Spearman’s rank correlation coefficients (r value). (1) |r| < 0.1, trivial; (2) 0.1 ≤ |r| ≤ 0.29, weak; (3) 0.3 ≤ |r| ≤ 0.49, moderate; (4) |r| ≥ 0.5, strong. Abbreviations: RAGO, robot-assisted gait orthosis. * *p* < 0.05, ** *p* < 0.01.

## Data Availability

The datasets used in the current study are available from the corresponding author on reasonable request.

## Supplementary Materials

The following supporting information can be downloaded at: https://www.mdpi.com/article/10.3390/ijerph19138186/s1, Figure S1. The RAGO training set up. The RAGO training used a body-weigh support (BWS) system to support body weight, electromechanical gait orthosis to drive legs on the sagittal plane of the treadmill, a mirror, and a screen with a display of the speed, force, and bilateral hip and knee motion to provide visual feedback regarding the gait pattern. Patient consent for publication of medical photography was obtained.

## Abbreviation

RAGO	Robot-assisted gait orthosis

## References

[1] Dettmann M.A., Linder M.T., Sepic S.B. (1987). Relationships among walking performance, postural stability, and functional assessments of the hemiplegic patient. Am. J. Phys. Med..

[2] Lai C.H., Chen H.C., Liou T.H., Li W., Chen S.C. (2019). Exercise interventions for individuals with neurological disorders: A systematic review of systematic reviews. Am. J. Phys. Med. Rehabil..

[3] Duncan P.W., Zorowitz R., Bates B., Choi J.Y., Glasberg J.J., Graham G.D., Katz R.C., Lamberty K., Reker D. (2005). Management of adult stroke rehabilitation care: A clinical practice guideline. Stroke.

[4] Li S., Francisco G.E., Zhou P. (2018). Post-stroke hemiplegic gait: New perspective and insights. Front. Physiol..

[5] Chen G., Patten C., Kothari D.H., Zajac F.E. (2005). Gait differences between individuals with post-stroke hemiparesis and non-disabled controls at matched speeds. Gait Posture.

[6] Hesse S. (2008). Treadmill training with partial body weight support after stroke: A review. NeuroRehabilitation.

[7] Hesse S.A., Jahnke M.T., Schreiner C., Mauritz K.H. (1993). Gait symmetry and functional walking performance in hemiparetic patients prior to and after a 4-week rehabilitation programme. Gait Posture.

[8] Srivastava A., Taly A.B., Gupta A., Kumar S., Murali T. (2016). Bodyweight-supported treadmill training for retraining gait among chronic stroke survivors: A randomized controlled study. Ann. Phys. Rehabil. Med..

[9] Drużbicki M., Przysada G., Guzik A., Brzozowska-Magoń A., Kołodziej K., Wolan-Nieroda A., Majewska J., Kwolek A. (2018). The efficacy of gait training using a body weight support treadmill and visual biofeedback in patients with subacute stroke: A randomized controlled trial. BioMed Res. Int..

[10] Belda-Lois J.M., Mena-del Horno S., Bermejo-Bosch I., Moreno J.C., Pons J.L., Farina D., Iosa M., Molinari M., Tamburella F., Ramos A. (2011). Rehabilitation of gait after stroke: A review towards a top-down approach. J. Neuroeng. Rehabil..

[11] Rodrigues T.A., Goroso D.G., Westgate P.M., Carrico C., Batistella L.R., Sawaki L. (2017). Slow Versus fast robot-assisted locomotor training after severe stroke: A randomized controlled trial. Am. J. Phys. Med. Rehabil..

[12] Kubota S., Nakata Y., Eguchi K., Kawamoto H., Kamibayashi K., Sakane M., Sankai Y., Ochiai N. (2013). Feasibility of rehabilitation training with a newly developed wearable robot for patients with limited mobility. Arch. Phys. Med. Rehabil..

[13] Lünenburger L., Colombo G., Riener R., Dietz V. Biofeedback in gait training with the robotic orthosis Lokomat. Proceedings of the Annual International Conference of the IEEE Engineering in Medicine and Biology Society IEEE Engineering in Medicine and Biology Society Annual Conference.

[14] Schück A., Labruyère R., Vallery H., Riener R., Duschau-Wicke A. (2012). Feasibility and effects of patient-cooperative robot-aided gait training applied in a 4-week pilot trial. J. Neuroeng. Rehabil..

[15] Yang H.E., Kyeong S., Lee S.H., Lee W.J., Ha S.W., Kim S.M., Kang H., Lee W.M., Kang C.S., Kim D.H. (2017). Structural and functional improvements due to robot-assisted gait training in the stroke-injured brain. Neurosci. Lett..

[16] Swinnen E., Beckwee D., Meeusen R., Baeyens J.P., Kerckhofs E. (2014). Does robot-assisted gait rehabilitation improve balance in stroke patients? A systematic review. Top. Stroke Rehabil..

[17] Mayr A., Kofler M., Quirbach E., Matzak H., Frohlich K., Saltuari L. (2007). Prospective, blinded, randomized crossover study of gait rehabilitation in stroke patients using the Lokomat gait orthosis. Neurorehabilit. Neural Repair.

[18] Hsu C.Y., Cheng Y.H., Lai C.H., Lin Y.N. (2019). Clinical non-superiority of technology-assisted gait training with body weight support in patients with subacute stroke: A meta-analysis. Ann. Phys. Rehabil. Med..

[19] Husemann B., Muller F., Krewer C., Heller S., Koenig E. (2007). Effects of locomotion training with assistance of a robot-driven gait orthosis in hemiparetic patients after stroke: A randomized controlled pilot study. Stroke.

[20] Hidler J., Nichols D., Pelliccio M., Brady K., Campbell D.D., Kahn J.H., Hornby T.G. (2009). Multicenter randomized clinical trial evaluating the effectiveness of the Lokomat in subacute stroke. Neurorehabilit. Neural Repair.

[21] Pohl M., Werner C., Holzgraefe M., Kroczek G., Mehrholz J., Wingendorf I., Hoolig G., Koch R., Hesse S. (2007). Repetitive locomotor training and physiotherapy improve walking and basic activities of daily living after stroke: A single-blind, randomized multicentre trial (DEutsche GAngtrainerStudie, DEGAS). Clin. Rehabil..

[22] Schwartz I., Sajin A., Fisher I., Neeb M., Shochina M., Katz-Leurer M., Meiner Z. (2009). The effectiveness of locomotor therapy using robotic-assisted gait training in subacute stroke patients: A randomized controlled trial. PM R J. Inj. Funct. Rehabil..

[23] Hornby T.G., Campbell D.D., Kahn J.H., Demott T., Moore J.L., Roth H.R. (2008). Enhanced gait-related improvements after therapist- versus robotic-assisted locomotor training in subjects with chronic stroke: A randomized controlled study. Stroke.

[24] Aprile I., Iacovelli C., Padua L., Galafate D., Criscuolo S., Gabbani D., Cruciani A., Germanotta M., Di Sipio E., De Pisi F. (2017). Efficacy of Robotic-Assisted Gait Training in chronic stroke patients: Preliminary results of an Italian bi-centre study. NeuroRehabilitation.

[25] Westlake K.P., Patten C. (2009). Pilot study of Lokomat versus manual-assisted treadmill training for locomotor recovery post-stroke. J. Neuroeng. Rehabil..

[26] Bruni M.F., Melegari C., De Cola M.C., Bramanti A., Bramanti P., Calabro R.S. (2018). What does best evidence tell us about robotic gait rehabilitation in stroke patients: A systematic review and meta-analysis. J. Clin.Neurosci. Off. J. Neurosurg. Soc. Australas..

[27] Bang D.H., Shin W.S. (2016). Effects of robot-assisted gait training on spatiotemporal gait parameters and balance in patients with chronic stroke: A randomized controlled pilot trial. NeuroRehabilitation.

[28] Knaepen K., Mierau A., Swinnen E., Fernandez Tellez H., Michielsen M., Kerckhofs E., Lefeber D., Meeusen R. (2015). Human-robot interaction: Does robotic guidance force affect gait-related brain dynamics during robot-assisted treadmill walking?. PLoS ONE.

[29] Khaw J., Subramaniam P., Abd Aziz N.A., Ali Raymond A., Wan Zaidi W.A., Ghazali S.E. (2021). Current update on the clinical utility of MMSE and MoCA for stroke patients in Asia: A systematic review. Int. J. Environ. Res. Public Health.

[30] Folstein M.F., Folstein S.E., McHugh P.R. (1975). “Mini-mental state”. A practical method for grading the cognitive state of patients for the clinician. J. Psychiatr. Res..

[31] Berg K.O., Wood-Dauphinee S.L., Williams J.I., Maki B. (1992). Measuring balance in the elderly: Validation of an instrument. Can. J. Public Health Rev. Can. De Sante Publique.

[32] Wang T.Y., Chen S.C., Peng C.W., Kang C.W., Chen Y.L., Chen C.L., Chou Y.L., Lai C.H. (2017). Relevance of nerve conduction velocity in the assessment of balance performance in older adults with diabetes mellitus. Disabil. Rehabil..

[33] Yuan R.Y., Chen S.C., Peng C.W., Lin Y.N., Chang Y.T., Lai C.H. (2020). Effects of interactive video-game-based exercise on balance in older adults with mild-to-moderate Parkinson’s disease. J. Neuroeng. Rehabil..

[34] Berg K., Wood-Dauphinee S., Williams J.I. (1995). The balance scale: Reliability assessment with elderly residents and patients with an acute stroke. Scand. J. Rehabil. Med..

[35] Cameron D., Bohannon R.W. (2000). Criterion validity of lower extremity motricity index scores. Clin. Rehabil..

[36] Perry J., Garrett M., Gronley J.K., Mulroy S.J. (1995). Classification of walking handicap in the stroke population. Stroke.

[37] Quinn T.J., Langhorne P., Stott D.J. (2011). Barthel index for stroke trials: Development, properties, and application. Stroke.

[38] Shah S.K., Harasymiw S.J., Stahl P.L. (1986). Stroke rehabilitation: Outcome based on brunnstrom recovery stages. OTJR Occup. Particip. Health.

[39] Kelley C.P., Childress J., Boake C., Noser E.A. (2013). Over-ground and robotic-assisted locomotor training in adults with chronic stroke: A blinded randomized clinical trial. Disabil. Rehabil. Assist. Technol..

[40] Chisari C., Bertolucci F., Monaco V., Venturi M., Simonella C., Micera S., Rossi B. (2015). Robot-assisted gait training improves motor performances and modifies Motor Unit firing in poststroke patients. Eur. J. Phys. Rehabil. Med..

[41] Harris N.K., Cronin J.B., Hopkins W.G., Hansen K.T. (2008). Relationship between sprint times and the strength/power outputs of a machine squat jump. J. Strength Cond. Res..

[42] Hidler J., Sainburg R. (2011). Role of Robotics in Neurorehabilitation. Top. Spinal Cord Inj. Rehabil..

[43] van Kammen K., Boonstra A.M., van der Woude L.H., Reinders-Messelink H.A., den Otter R. (2016). The combined effects of guidance force, bodyweight support and gait speed on muscle activity during able-bodied walking in the Lokomat. Clin. Biomech..

[44] Krishnan C., Kotsapouikis D., Dhaher Y.Y., Rymer W.Z. (2013). Reducing robotic guidance during robot-assisted gait training improves gait function: A case report on a stroke survivor. Arch. Phys. Med. Rehabil..

[45] Conditt M.A., Gandolfo F., Mussa-Ivaldi F.A. (1997). The motor system does not learn the dynamics of the arm by rote memorization of past experience. J. Neurophysiol..

[46] Tyson S.F., Woodward-Nutt K., Plant S. (2018). How are balance and mobility problems after stroke treated in England? An observational study of the content, dose and context of physiotherapy. Clin. Rehabil..

[47] Lee S.Y., Han E.Y., Kim B.R., Chun M.H., Lee Y.K. (2017). Can lowering the guidance force of robot-assisted gait training induce a sufficient metabolic demand in subacute dependent ambulatory patients with stroke?. Arch. Phys. Med. Rehabil..

[48] van Kammen K., Boonstra A.M., van der Woude L.H.V., Visscher C., Reinders-Messelink H.A., den Otter R. (2020). Lokomat guided gait in hemiparetic stroke patients: The effects of training parameters on muscle activity and temporal symmetry. Disabil. Rehabil..

[49] McGrath R.L., Ziegler M.L., Pires-Fernandes M., Knarr B.A., Higginson J.S., Sergi F. (2019). The effect of stride length on lower extremity joint kinetics at various gait speeds. PLoS ONE.

