# Supplementary material for: Adjustable Parameters and the Effectiveness of Adjunct Robot-Assisted Gait Training in Individuals with Chronic Stroke
Source: Int J Environ Res Public Health. 2022 Jul 4;19(13):8186. doi: 10.3390/ijerph19138186 (PMC9265951; doi:10.3390/ijerph19138186)
Supplement: Supplementary file 1 [file ijerph-19-08186-s001.zip › ijerph-1713718-supplementary.pdf]

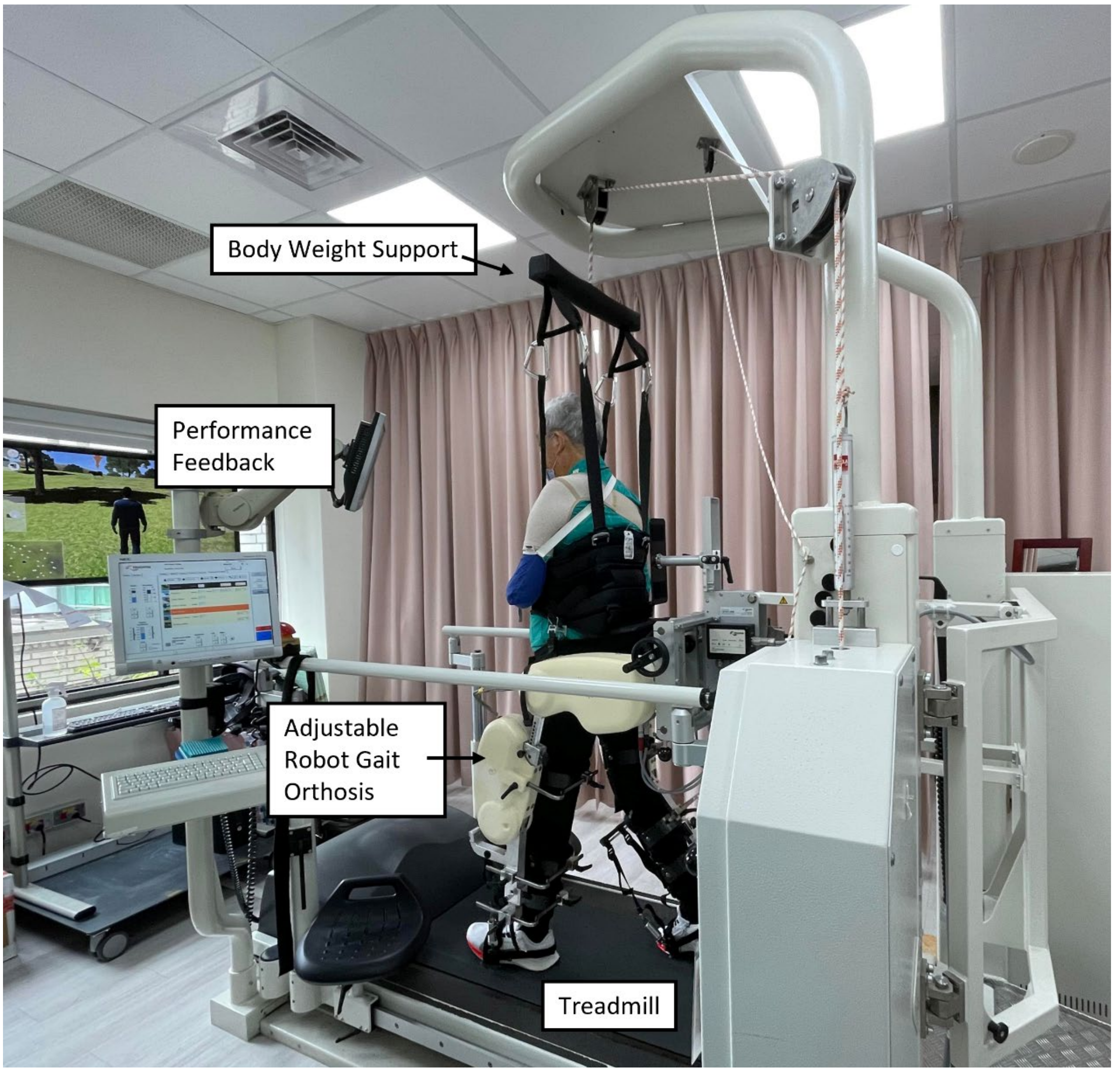

Figure S1. The RAGO training set up. The RAGO training used a body-weigh support (BWS) system to support body weight, electromechanical gait orthosis to drive legs on the sagittal plane of the treadmill, a mirror, and a screen with a display of the speed, force, and bilateral hip and knee motion to provide visual feedback regarding the gait pattern. Patient consent for publication of medical photography was obtained.
